# Treatment of high-energy pilon fractures using the ILIZAROV treatment

**DOI:** 10.11604/pamj.2017.27.199.11066

**Published:** 2017-07-14

**Authors:** Walid Osman, Zeineb Alaya, Hamdi Kaziz, Lassad Hassini, Meriem Braiki, Nader Naouar, Mohamed Laaziz Ben Ayeche

**Affiliations:** 1 Department of Orthopedic Surgery, University College Hospital Sahloul, Tunisia; 2 Department of Rheumatology, University College Hospital Farhat Hached, Tunisia

**Keywords:** Pilon fracture, distal tibia, external fixation, ilizarov external fixator

## Abstract

The management of high-energy pilon fractures is still controversial. Open reduction and internal fixation are often associated with serious complications. Various methods have been used to treat these injuries, with variable results. The aim of this retrospective study was to analyze the clinical and radiographic outcome of the ILIZAROV technique in patients with high-energy pilon fractures. Thirty cases of distal tibia epiphysis fractures (pilon fractures) were managed from 1999 to 2012. The study group included 5 cases of open fractures. The mean age was 47 years. According to Rüedi and Algower classification; 11 fractures were type II, and 19 type III. All fractures were a consequence of high-energy trauma. Fractures of the lower fibula were present in 28 of the patients. An external Fixator was applied for open fractures. Closed injuries were operated on 3 to 13 days after injury, with an average of 8 days. The mean follow-up was 48 months. All fractures united. The external fixator was removed after a mean of 22 weeks (10 - 28 weeks). Two patients with a type III fracture had a delayed union and were treated with corticotomy and dynamisation of the ILIZAROV fixator. Only one secondary displacement of a type III fracture was noted after two months and was treated by adjuction of 2 olive wires. There were no cases of osteomyelitis or deep infections. Pin-tract infections occurred in ten patients. We had not any case of nervous injury due to introduction of the pins. Using radiological criteria for assessement of reduction of the articular fragments, there was excellent and good restoration of articular structure in 24 cases. The average American Orthopeadic Foot and Ankle Society ankle-hind foot score was excellent in 16, good in 6, fair in 6 and poor in 2. Soft tissue healing occurred without need for plastic surgery in all cases. The movements of the ankle ranged from 0 to 20° of dorsiflexion and 5° to 40° of plantar flexion. Twenty patients had gone back to their preinjury profession. The ILIZAROV technique is a safe and a very effective treatment for severe pilon fractures with minimum complications and good healing results.

## Introduction

Tibial pilon fractures affect the bottom of the tibia (shinbone) at the ankle joint. Tibial pilon fractures constitute 1% of all lower extremity fractures and are one of the most difficult fracture types to manage [1]. Motor vehicle accidents and falls from a height are the most frequent injury mechanisms. Most tibial pilon fractures are the result of axial loading, and fast axial loading disseminates an excessive amount of energy. This energy release causes severe soft tissue injuries in tibial fractures [2]. Since the development of external fixators, their minimal soft tissue damage have made these techniques increasingly popular [2]. In this retrospective study, we aimed to present the clinical and radiological outcomes of patients with complex tibial pilon fractures who were treated with ring external fixators in order to evaluate the efficiency of this approach in the management of such complex conditions.

## Methods

**Figure 1:**
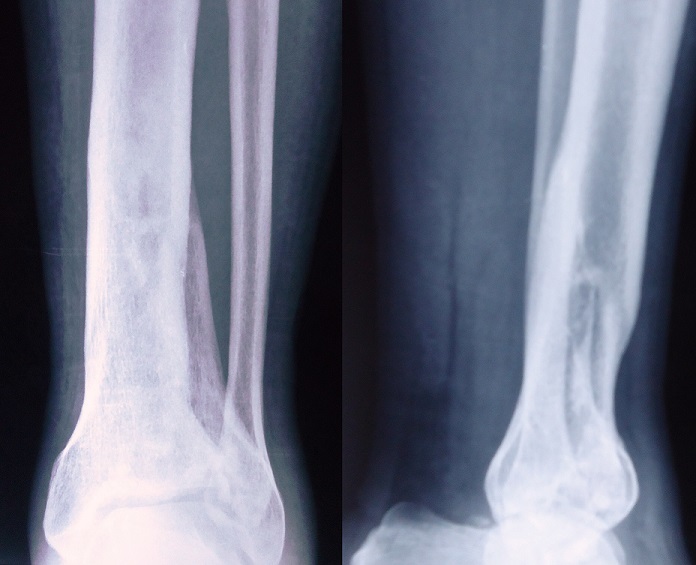
Preoperative anteroposterior and lateral view shows the Ruedi type II pilon fracture

**Figure 2:**
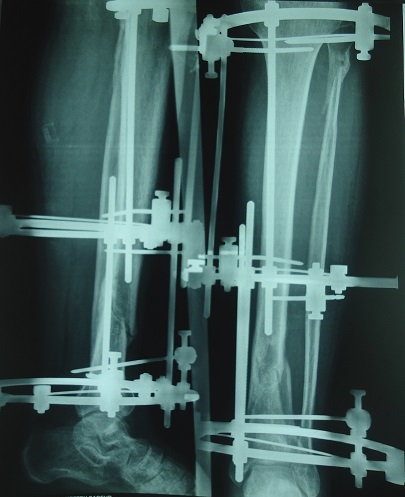
Immediately postoperative X-ray shows good reduction of the pilon fracture

Patients who underwent surgical repair for complex tibial pilon fractures between 1999 and 2012 were evaluated retrospectively via electronic medical records. The study was conducted in the orthopedic department of the University Hospital Sahloul in Sousse. The mean age of patients was 47 years (20-73years) including 26 men and 4 women. All fractures were due to a high-energy trauma. 15 patients had sustained leg trauma in road traffic accidents, while the remaining had fallen from a height. Five of the patients had open fractures (three type I and 2 type II). In cases of open fractures, the wound was debrided early, repeated after 48-72 h if necessary. Skin injuries as phlyctena or contusion were present in 5 cases. According to Rüedi and Algower classification [3]; 11 fractures were type II (Figure 1), and 19 type III. The surgical management involved stabilization by the diaphyseal-epiphyseal ILIZAROV technique of external fixation (Figure 2). Fractures of the lower fibula were present in 28 of the patients. All open fractures were treated with wound debridement and application of a ring fixator. Closed injuries were operated on 3 to 13 days after injury, with an average of 8 days. ILIZAROV devices remained in place for an average of 12 weeks. The mean follow-up period was of 48 months (12-76 months). Fractures healing was followed after the operation. The American foot and ankle society (AOFAS) scoring system was used for clinical evaluation [3].

### Surgical technique

Under general anesthesia, patients were placed in the supine position with transcalcaneal traction. Biplane fluoroscopy was used during reduction, pin insertion and assembly of the frame. The quality of reduction, achieved by ligamentotaxis, was checked using intensification. Internal fixation was initially required for the lateral malleolus to restore fibular length. The frame was made of two stainless steel rings for diaphyseal fixation and a single radiolucent carbon fiber ring for the epiphysis. The fracture was reduced with the traction and manual pressure. When optimal anatomical reduction was obtained and confirmed by fluoroscopy. The pins were placed to stabilize the anteromedial and anterolateral fragments to each other. Once reduction of the anterior fragments is accomplished, displacement of the posterior malleolus is achieved with dorsiflexion of the foot. The reduction is maintained with a guide wire for subsequent insertion of a cannulated screw. Then, the fragments could be manipulated percutaneously with a joker. When the articular surface was severely comminuted, visualization of the articular surface was necessary and was accomplished through a 2cm longitudinal anterior incision for the joint exposure. The metaphyseal fracture site was not exposed. The circular frame is then assembled on that wire. The wires are fixed to the rings of the fixator and tensioned. When necessary the foot is incorporated by wires inserted in the calcaneus and fixed to a half ring connected to the tibial external fixator. The foot is fixed in neutral position to avoid supination and equines position. Active and passive range-of-motion exercises were begun immediately after surgery with no restriction on weight-bearing.

## Results

**Figure 3:**
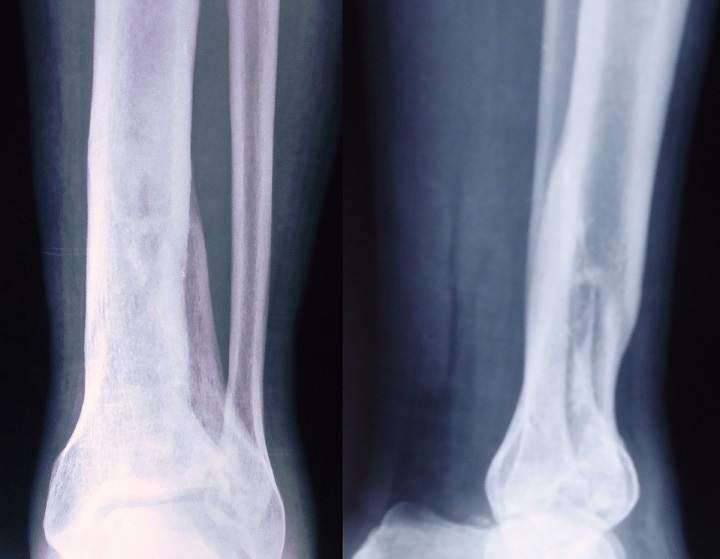
Follow-up radiographs after 42 months, showing good fracture union

**Figure 4:**
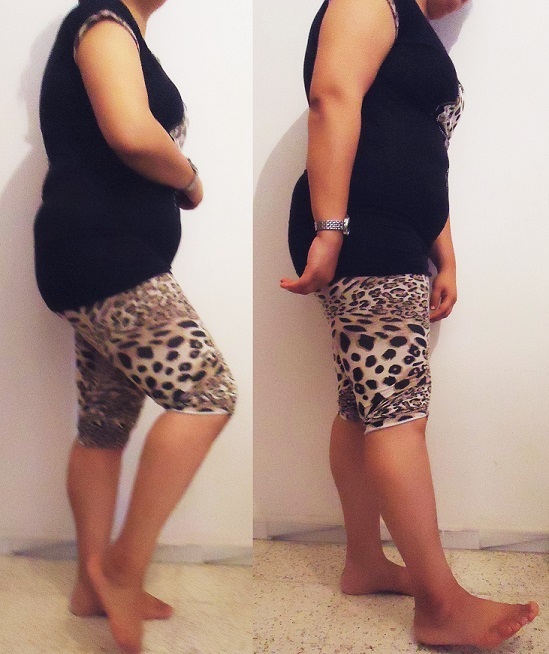
Follow-up clinical outcome showing excellent functional outcome with ability to squat

The mean age of the population was 47 years (range, 20-73years), and the mean follow-up interval was 48 months. All patients experienced tibial pilon fractures as a result of high energy trauma. The assessement of articular fragments reduction was made according to criteria described by Ovadia and beals (Table 1). Clinical outcomes were evaluated according to the ankle-hindfoot score devised by the American Orthopaedic Foot and Ankle Society (AOFAS) (Table 2) [3]. Reduction was judjed good in 10 patients, fair in 14, and poor in 6 (Table 1). Soft tissue healing occurred without need for plastic surgery in all cases. The external fixator was removed after a mean of 22 weeks (range 10 - 28 weeks). Two patients with a type III fracture experienced delayed union treated with corticotomy and dynamisation of the ILIZAROV apparatus. Consolidation was obtained in a mean of 3 months (Figure 3). Ten cases of local infections of pins have been treated by antibiotics and local antiseptic. We had not any case of nervous injury due to introduction of the pins. Only one secondary displacement of a type III fracture was noted after two months and was treated by adjuction of 2 olive wires. Despite adequate external reduction, 4 malunion occurred. In a varus-valgus plane, the final alignement was neutral +-5° for 27 fractures. One fracture had 20° valgus malalignement; ankle fusion was eventually performed. A 5° valgus deformity also occurred after ILIZAROV apparatus removal in a 28 years woman with a type III fracture. A short cast leg was applied for 30 days and the final clinical result was judjed good. In a flexion/extension plane, the final alignement was neutral ± 5° for all 26 fractures. There was radiological evidence of arthritis in eleven of the patients leading to ankle arthrodesis in one case. Clinical results according to the AOFS score was excellent in 16 patients (Figure 4), good in 6 patients, fair in 6 and poor in 2. Eighteen patients were satisfied with the surgery. Sixteen of the patients had ankle pain. The movements of the ankle ranged from 0 to 20° of dorsiflexion and 5° to 40° of plantar flexion. Twenty patients had gone back to their preinjury profession.

**Table 1:** Quality of reduction

Anatomic	8 points	5 cases
Good	9-11 points	15 cases
Fair	12-15 points	6 cases
Poor	> 15 points	4 cases

**Table 2:** Subjective part of the AOFAS score (maximum 60 points)

**Pain (maximum 40 points)**	
None	40 points
Mild, occasional	30 points
Moderate, daily	0 points
Severe, almost always present	0 points
**Function (maximum 10 points)**	
No activity limitations, no support needed	10 points
No limitations of daily activities, limitation of recreational activities, no support	7 points
Limited daily and recreational activities, cane	4 points
Severe limitation of daly and recreational activities, walker, cruches, wheelchair, brace	0 points
**Maximum walking distance (maximum 5 points)**	
Greater than 6 blocks	5 points
4-6 blocks	4 points
1-3 blocks	2 points
Less than 1 blocks	0 points
**Walking surfaces (maximum 5 points)**	
No difficulty on any surface	5 points
Some difficulty on uneven terrain, stairs, inclines, ladders	3 points
Severe difficulty on uneven terrain, stairs, inclines, ladders	0 points

## Discussion

Tibial pilon fractures are complex and difficult to treat. They represent about 1% of all fractures of the lower extremities, and up to 10% of the tibial fractures [4]. The damage is caused by high-energy trauma mainly in axial load as the usual consequence of road accidents or falls from a considerable height [4]. The main objectives of the treatment of tibial pilon fractures are the maintenance of length, recreation of the joint surfaces and restoration of limb alignment. Open reduction provides the safest way of achieving fracture reduction and restoring joint congruity, considering also the possibility of external fixation to restore severe articular comminution in type C fractures (AO/ OTA) [5].

Surgical methods may be used, such as external fixation, the intramedullary nail, the percutaneous synthesis with cannulated wires or Kirschner's wires and a synthesis with modern plates [6]. Surgical options are internal fixation, external fixation with or without limited internal fixation and primary arthrodesis [4]. There is a high rate of infection and wound sloughing following open reduction and internal fixation of pilon fractures. The risk of arthrodesis or amputation has caused many surgeons to attempt reconstruction of the articular surface without exposing the fracture [7, 8]. The use of the external fixator in treating pilon fractures allows restoration of length, stabilization of the limb, and correction of the mechanical axis. In fact, with the external fixator used as a neutralization device, there is no need for large plates with the increased risk of infection and skin sloughing [7].

The most significant advantage of ring fixators is their ability to achieve stability with minimal soft-tissue dissection [6, 9]. A circular fixator also provides the option of bone transport. The Ilizarov fixator is superior in strength and stability to the hybrid type and allows early weight-bearing. This helps in preventing peri-articular osteopenia, and the tensioned wires allow axial micromovement, which is known to promote bony union [6]. Mitkovic et al [10], described 26 patients with 28 Type C3, distal intraarticular tibial (pilon) fractures treated by dynamic external fixation. Follow-up was at least two years, and the results (subjective and objective) were classified according to the Ovadia system, they found 71% subjectively and 67% objectively excellent results. The mean to fracture union was 14 weeks (range: 12-20 weeks). There were three cases with angulation deformity (from 7 to 20) [11]. There were no cases with nonunion or deep infection despite a high frequency of infections (11%) and osteoarthritis (15%). Based on these results, this treatment with closed reduction and dynamic external fixation allowing early motion appears as a suitable method for treatment of comminuted intraarticular tibial pilon fractures [4]. Watson et al [12], described 107 pilon fractures, of which 36 were treated with ORIF and 58 with external fixation. They observed late complications such as osteoarthritis and arthrodesis in both groups, but the incidence of unplanned secondary procedures was higher in the ORIF group, which the authors attributed to extensive stripping of soft tissue during open reduction. They recommended external fixation for high-energy pilon fractures [6].

External fixation is a recognised alternative treatment for high-energy pilon fractures [6]. McDonald et al [13], observed no deep infection or osteomyelitis with a high rate of fracture healing and good functional recovery after external stabilisation of 13 pilon fractures, 12 of which were Ruedi type II or III. Marsh et al [14], observed no wound breakdown and only two cases of infection of the fibular plating site. Bone et al [15], in a study of 20 high energy open pilon fractures managed with ankle spanning external fixators, found that these fractures are better managed by external fixation with or without minimal internal fixation than with plate osteosynthesis. They had no infections. Another study of Golubovi et al [16] included 47 patients with tibial pilon fractures (33 (70.2%) males and 14 (29.8%) females). The patients mean age was 45.8 years. In the first group, which consisted of 22 patients, open reduction and internal fixation of both the tibia and the fibula was performed in the two separate incisions. The second group consisted of 25 patients managed with external fixation by external fixator. The patients were followed-up inside a 24-month-period. The obtained was a substantially high number of complications after open reduction and internal fixation in the first group of patients [16]. Lovisetti et al [17] reported a study of 30 cases of AO type 43 C tibial fractures treated by transouseous osteosynthesis (ILIZAROV technique). There were excellent and good restoration of articular structure in 27 cases and good clinical results in 15 cases. There was no case of pseudarthrosis or deep infection. Union occurred in all cases. A retrosprective study of 21 patients with high energy tibial pilon fractures treated with ILIZAROV technique, Vidyadhara et al [11] found encouraging results with good functional outcome in 76% patients. There were no-long-term problems with fracture union, and no patient required an ankle arthrodesis.

In our group study, all fractures united. We attribute the 100% union rate to meticulous respect of soft tissue envelope made possible by the strategy of treatment. Despite the quality of reduction achieved and lack of complications observed, the clinical outcomes in our series have been less favorable than others; there were excellent and good functional results in 73%. It is believed that approximately 10° of dorsiflexion of the ankle is required for an adequate functional gait [6]. We achieved a relatively higher functional ankle range of motion with 5° to 40° of plantar flexion and 0° to 20° of dorsiflexion. However, pin bottom infection was observed in 10 patients in our study group. Antibiotic therapy was administered to these patients with a good recovery.

At the latest follow-up evaluation, eleven patients developed ankle arthritis and the results can not be compared with any other study in the literature because of the fractures in this series were a consequence of high-energy trauma. This may indicate that outcomes may not always parallel to the quality of reduction achieved as assessed by radiograph. In this study, the Ilizarov fixator is found to be an excellent method in treating pilon fractures especially when adequately applied.

Thus, Tensional small-wire fixation gives good stability to reduced fracture fragments, none of the patient had loss of fixation. The use of olive wires in opposition helps compression of fracture fragments. The ILIZAROV percutaneous fixator preserves endosteal and periosteal blood supply, helps capture the small metaphyseal and subchondral bony fragments, and also helps compression of fracture fragments using the olive wires. The rigidity of fixation can be adjusted to suit stage of fracture healing. It also allows correction of deformity during the process of fracture healing. Early mobilization of the ankle joint is another advantage of the ILIZAROV device.

## Conclusion

The authors concluded that it is possible to achieve a satisfactory outcome, in pilon fractures, with the ILIZAROV technique allowing early definitive treatment and unrestricted weight bearing. It is these complex fractures that the external fixator was effective in that did not involve many of the complications associated with other methods of treatment because it allows for flexibility in obtaining neutral alignement and preserves soft tissues. Satisfactory functional reconstruction with fairly good functional results in most patients, with a moderate periarticular complication rate, have led us to use this treatment in these comminuted pilon fractures.

### What is known about this topic

- Tibial pilon fractures are complex and difficult to treat;
- Surgical options are internal fixation, external fixation with or without limited internal fixation and primary arthrodesis;
- There is a high rate of infection and wound sloughing following open reduction and internal fixation of pilon fractures.

### What this study adds

- The ILIZAROV technique is a safe treatment for severe pilon fractures with minimum complications;
- The ILIZAROV technique is a very effective treatment for severe pilon fractures with good healing results.

## Competing interests

The authors declare no competing interest.

## References

[1] Villaseñor Villaseñor LE, Olea Leyva MA, Rodríguez Flores R, Hernández López JL (2009). Clinical outcome of a bilateral tibial pylon fracture treated with a minimally invasive technique. Acta Ortop Mex..

[2] Bülbül M, Kuyucu E, Say F, Kara A, Erdil M (2015). Hybrid external fixation via a minimally invasive method for tibial pilon fractures -Technical note. Ann Med Surg (Lond).

[3] Kitaoka HB, Alexander IJ, Adelaar RS, Nunley JA, Myerson MS, Sanders M (1994). Clinical rating systems for the ankle-hindfoot, midfoot, hallux and lesser toes. Foot Ankle Int..

[4] Galante VN, Vicenti G, Corina G, Mori C, Abate A, Picca G, Conserva V, Speciale D, Scialpi L, Tartaglia N, Caiaffa V, Moretti B (2016). Hybrid external fixation in the treatment of tibial pilon fractures: A retrospective analysis of 162 fractures. Injury..

[5] Mauffrey C, Vasario G, Battiston B, Lewis C, Beazley J, Seligson D (2011). Tibial pilon fractures: a review of incidence, diagnosis, treatment, and complications. Acta Orthop Belg.

[6] Kapoor SK, Kataria H, Patra SR, Boruah T (2010). Capsuloligamentotaxis and definitive fixation by an ankle-spanning Ilizarov fixator in high-energy pilon fractures. J Bone Joint Surg Br.

[7] El-Mowafi H, El-Hawary A, Kandil Y (2015). The management of tibial pilon fractures with the Ilizarov fixator: the role of ankle arthroscopy. Foot (Edinb)..

[8] Bourne RB, Rorabeck CH, Macnab J (1983). Intra-articular fractures of the distal tibia:the pilon fracture. J Trauma..

[9] Beals TC (2001). Application of ring fixators in complex foot and ankle trauma. Orthop Clin North Am..

[10] Mitkovic MB, Bumbasirevic MZ, Lesic A, Golubovic Z (2002). Dynamic external fixation of comminuted intra-articular fractures of the distal tibia (type C pilon fractures). Acta Orthop Belg..

[11] Vidyadhara S, Rao SK (2006). Ilizarov treatment of complex tibial pilon fractures. Int Orthop..

[12] Watson JT, Moed BR, Karges DE, Cramer KE (2000). Pilon fractures: treatment protocol based on severity of soft tissue injury. Clin Orthop Relat Res..

[13] McDonald MG, Burgess RC, Bolano LE, Nicholls PJ (1996). Ilizarov treatment of pilon fractures. Clin Orthop Relat Res..

[14] Marsh JL, Bonar S, Nepola JV, Decoster TA, Hurwitz SR (1995). Use of an articulated external fixator for fractures of the tibial plafond. J Bone Joint Surg Am..

[15] Bone L, Stegemann P, McNamara K, Seibel R (1993). External fixation of severely comminuted and open tibial pilon fractures. Clin Orthop Relat Res..

[16] Golubović Z, Macukanović-Golubović L, Stojiljković P, Jovanović J, Micić I, Stojiljkovioć D, Milić D, Mitković M (2007). External fixation combined with limited internal fixation in the treatment of pilon tibia fractures. Vojnosanit Pregl..

[17] Tornetta P, Weiner L, Bergman M, Watnik N, Steuer J, Kelley M, Yang E (1993). Pilon fractures: treatment with combined internal and external fixation. J Orthop Trauma..

